# Dihydroflavonol 4-Reductase Genes Encode Enzymes with Contrasting Substrate Specificity and Show Divergent Gene Expression Profiles in *Fragaria* Species

**DOI:** 10.1371/journal.pone.0112707

**Published:** 2014-11-13

**Authors:** Silvija Miosic, Jana Thill, Malvina Milosevic, Christian Gosch, Sabrina Pober, Christian Molitor, Shaghef Ejaz, Annette Rompel, Karl Stich, Heidi Halbwirth

**Affiliations:** 1 Vienna University of Technology, Institute of Chemical Engineering, Vienna, Austria; 2 Institut für Biophysikalische Chemie, Fakultät für Chemie, Universität Wien, Vienna, Austria; 3 Bahauddin Zakariya University, Department of Horticulture, Multan, Pakistan; University of Iowa, United States of America

## Abstract

During fruit ripening, strawberries show distinct changes in the flavonoid classes that accumulate, switching from the formation of flavan 3-ols and flavonols in unripe fruits to the accumulation of anthocyanins in the ripe fruits. In the common garden strawberry (*Fragaria*×*ananassa*) this is accompanied by a distinct switch in the pattern of hydroxylation demonstrated by the almost exclusive accumulation of pelargonidin based pigments. In *Fragaria vesca* the proportion of anthocyanins showing one (pelargonidin) and two (cyanidin) hydroxyl groups within the B-ring is almost equal. We isolated two dihydroflavonol 4-reductase (DFR) cDNA clones from strawberry fruits, which show 82% sequence similarity. The encoded enzymes revealed a high variability in substrate specificity. One enzyme variant did not accept DHK (with one hydroxyl group present in the *B-*ring), whereas the other strongly preferred DHK as a substrate. This appears to be an uncharacterized DFR variant with novel substrate specificity. Both *DFR*s were expressed in the receptacle and the achenes of both *Fragaria* species and the *DFR2* expression profile showed a pronounced dependence on fruit development, whereas *DFR1* expression remained relatively stable. There were, however, significant differences in their relative rates of expression. The *DFR1/DFR2* expression ratio was much higher in the *Fragaria*×*ananassa* and enzyme preparations from *F.*×*ananassa* receptacles showed higher capability to convert DHK than preparations from *F. vesca*. Anthocyanin concentrations in the *F.*×*ananassa* cultivar were more than twofold higher and the cyanidin:pelargonidin ratio was only 0.05 compared to 0.51 in the *F. ve*sca cultivar. The differences in the fruit colour of the two *Fragaria* species can be explained by the higher expression of *DFR1* in *F.*×*ananassa* as compared to *F. vesca*, a higher enzyme efficiency (*K*_cat_/*K*_m_ values) of DFR1 combined with the loss of F3’H activity late in fruit development of *F.*×*ananassa*.

## Introduction

The strawberry is an appealing plant model for studying flavonoid metabolism during fruit development, as there is not only a change in the flavonoid classes but also in their B-ring hydroxylation patterns. These hydroxylation patterns switch from mainly dihydroxylated flavan 3-ols and flavonols in unripe fruits to monohydroxylated anthocyanins in ripe fruits [1], [2], [3]. In the *F. vesca* the ratio of anthocyanins possessing one (pelargonidin type) and two (cyanidin type) hydroxyl groups in the *B-*ring is almost equal, whereas pelargonidin type anthocyanins are particularly prevalent in the *F.*×*ananassa*. This is frequently reflected in fruit colouration (Figure S1 in File S1) [1], [4]. The changes in the hydroxylation patterns can be achieved in two ways: either via the downregulation of *flavonoid 3′-hydroxylase* (*F3′H*) expression in late fruit stages or via the presence of a set of dihydroflavonol 4-reductases (DFR) showing different substrate specificities. Recently we have demonstrated that the differing hydroxylation pattern of anthocyanins in *F. vesca* and *F.*×*ananassa* is reflected in the *F3'H* expression pattern. In *F. vesca, F3'H* was highly expressed during all developmental stages. This contrasted sharply with a decline in the expression of *F3'H* observed in *F.*×*ananassa* [1], also reported for *anthocyanidin reductase* and *leucoanthocyanidin reductase,* genes specifically involved in flavan 3-ol formation [5]. To investigate whether DFR substrate specificity could also contribute to the establishment of strawberry fruit anthocyanin hydroxylation patterns, we studied DFR in two species of *Fragaria*.

**Figure 1:**
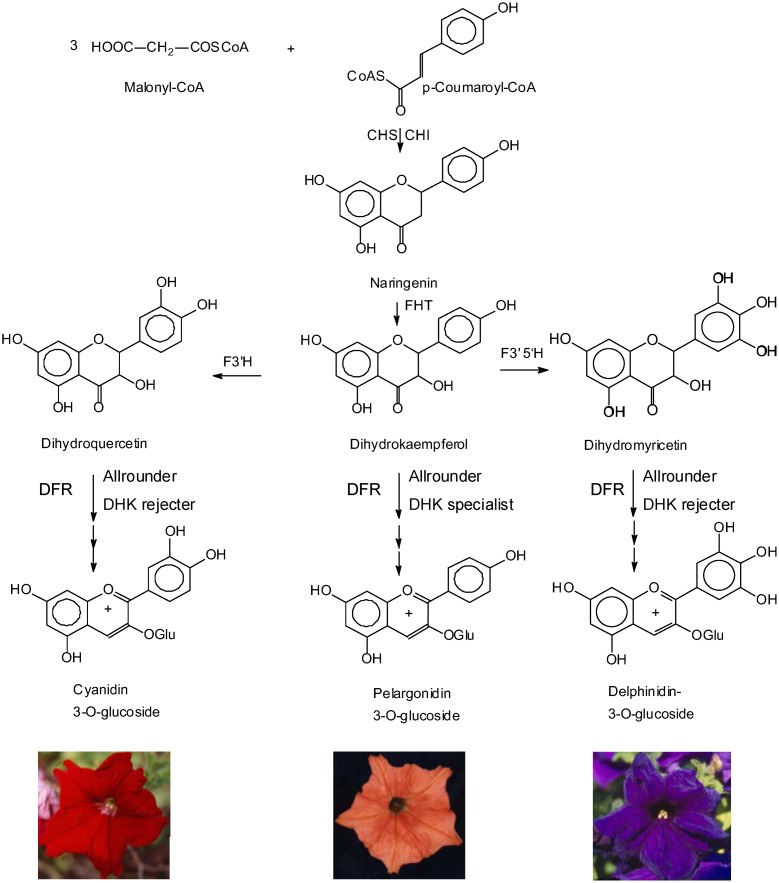
Simplified flavonoid pathway demonstrating the influence of DFR substrate acceptance on the establishment of flower colour.

DFR (EC 1.1.1.219) is an oxidoreductase which catalyzes the NADPH dependent reduction of the keto group in position 4 of dihydroflavonols to produce flavan 3,4-diols (synonym: leucoanthocyanidins), which are the immediate precursors for the formation of anthocyanidins and flavan 3-ols, the building blocks of condensed tannins [6]. DFR competes with flavonol synthase for dihydroflavonols as common substrates and therefore interferes with flavonol formation [7]. DFR thus has a strong influence on the formation of at least 3 classes of flavonoids, anthocyanin pigments, flavanols (which provide protection against herbivore, pests and pathogens), and flavonols (which act as sunscreens) [8], [9]. In addition, DFR is unique in the flavonoid pathway, because it can exhibit selectivity for the *B-*ring hydroxylation pattern of flavonoid substrates. While the DFRs of many plants accept dihydroflavonols possessing one (dihydrokaempferol, DHK), two (dihydroquercetin, DHQ) or three (dihydromyricetin, DHM) hydroxyl groups within the B-ring, specific DFRs have been described from a few plant species that do not convert DHK into the corresponding leucopelargonidin [10], [11]. These species do not produce pelargonidin based pigments and therefore lack an orange-red flower colouration. F3'H deficient lines for those species show a white or pale rosy flower colouration. Due to the absence of dihydroxylated precursors the formation of anthocyanins is not possible. A prominent example is petunia (*Petunia hybrida*) [10] where the biotechnological introduction of an non-specific DFR from maize bypassed a gap in the pathway of anthocyanin formation within the F3'H deficient petunia line RL01 [12], resulting in an orangered flower colouration (Figure 1, centre). To probe this phenomenon, an artificial DHK specific DFR was created via site-directed mutagenesis of the DFR from *Gerbera hybrida* with an exchange of an asparagine in position 134 into leucine [10]. This gene is patented for flower colour modification via transgenic approaches.

We report here on the isolation of two DFR cDNA clones from strawberry fruits demonstrating distinct but contrasting substrate specificity. The divergent ratio of expression between these DFRs in two *Fragaria* species contributes to the establishment of the different anthocyanin patterns within these fruits.

## Results and Discussion

### Cloning of DFR from *Fragaria* species

**Figure 2:**
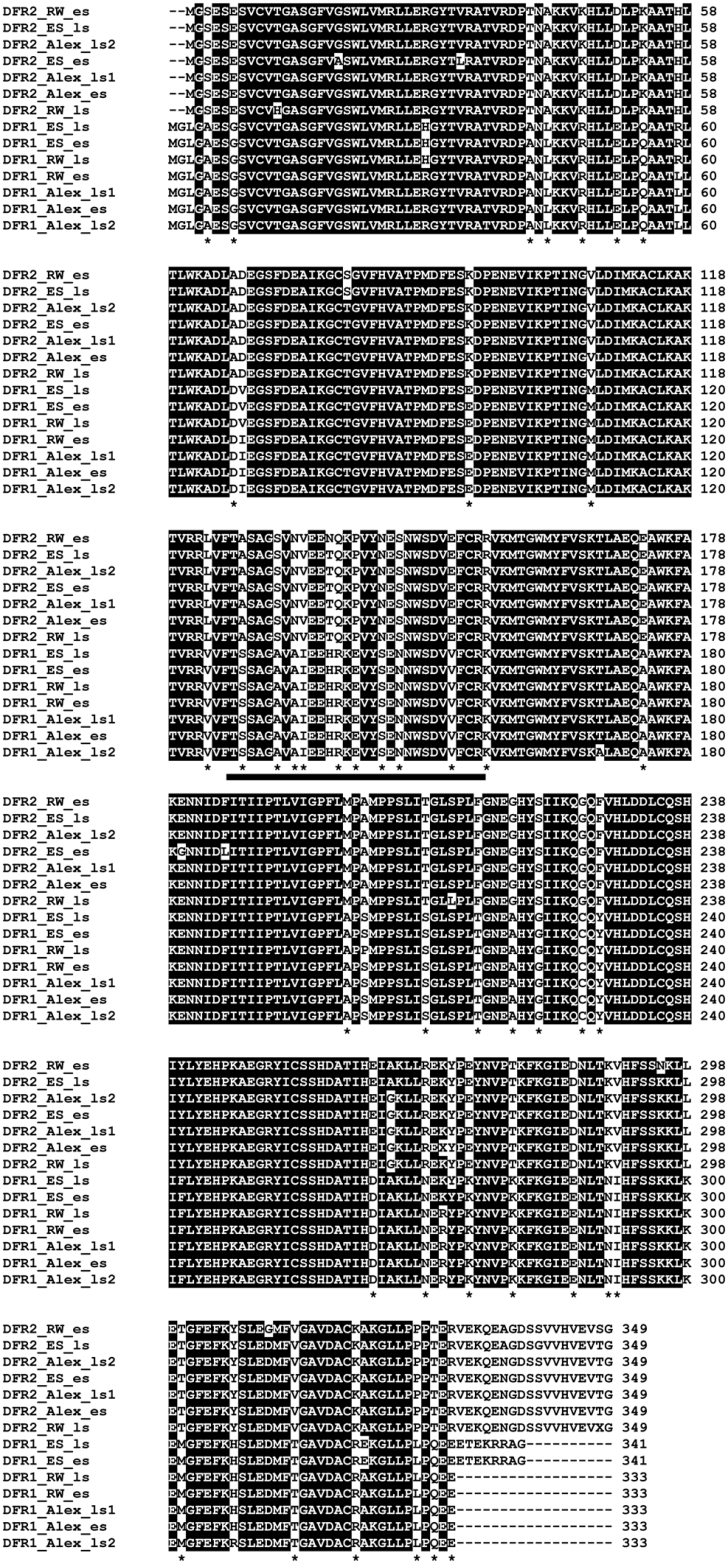
Multiple alignment of the deduced amino acid sequences encoded by *DFR*s isolated from *F. vesca* cv Alexandria during early (KC894042, KC894049) and late (KC894043, KC894044, KC894050, KC894051) stages, cv. Red Wonder early (KC894045, KC894052) and late (KC894046, KC894053) stages and *F*. ×***ananassa*** **cv. Elsanta early (KC894047, KC894054) and late (KC894048, KC894055) stages.** abbreviations: RW: Red Wonder, Alex: Alexandria, ES: Elsanta, es: early stage, ls: late stage.

The NCBI database lists eight *DFR* full-length clones (reference date March 31, 2014) from strawberry all presenting at least 95% sequence similarity with the first isolated *DFR* from *F*.×*ananassa* (Accession AF029685 [13]). To identify further putative *DFRs* we screened the *F*. *vesca* genome [14] for the presence of homologues of the well-known *DFR*. With this approach, a *DFR* sequence was found presenting only an 82% sequence identity. The paralogous genes were named *DFR1* (high similarity to Accession AF029685) and *DFR2* (so far unknown). Specific primers were designed for the two *DFR* variant and used for the isolation of cDNA clones from early and late developmental stages of fruits of *F*.×*ananassa* cv. Elsanta and *F*. *vesca* cv. Alexandria and cv. Red Wonder. 2–3 allelic variants of both *DFR* variants were isolated from each cultivar (GenBank IDs KC894042-KC894055, Table 1, Figure 2). *DFR1* consisted of 1026 bp with an open reading frame (ORF) of 341 deduced amino acids (*F*.×*ananassa*) and 999 bp with an ORF of 333 deduced amino acids (*F*. *vesca*), respectively. *DFR2* of both species consisted of 1050 bp with an ORF of 349 amino acids. *DFR1* and *DFR2* sequences shared 80–83% amino acid sequence identity. The sequence identity of *DFR2* was 97–99% between *F*. *vesca* and *F*.×*ananassa* and 98–100% between *F*. *vesca* cv. Alexandria and cv. Red Wonder. *DFR1* showed 96–98% sequence identity between *F*.×*ananassa* and *F*. *vesca*, and 97–100% between *F*. *vesca* cv. Alexandria and cv. Red Wonder.

**Table 1:** DFR cDNA clones isolated from *Fragaria* species.

DFR variant	Accession No	Species	cultivar	stage
DFR1	KC894042	*F*. *vesca*	Alexandria	early
DFR1	KC894043	*F*. *vesca*	Alexandria	late
DFR1	KC894044	*F*. *vesca*	Alexandria	late
DFR1	KC894045	*F*. *vesca*	Red Wonder	early
DFR1	KC894046	*F*. *vesca*	Red Wonder	late
DFR1	KC894047	*F*.×*ananassa*	Elsanta	early
DFR1	KC894048	*F*.×*ananassa*	Elsanta	late
DFR2	KC894049	*F*. *vesca*	Alexandria	early
DFR2	KC894050	*F*. *vesca*	Alexandria	late
DFR2	KC894051	*F*. *vesca*	Alexandria	late
DFR2	KC894052	*F*. *vesca*	Red Wonder	early
DFR2	KC894053	*F*. *vesca*	Red Wonder	late
DFR2	KC894054	*F*.×*ananassa*	Elsanta	early
DFR2	KC894055	*F*.×*ananassa*	Elsanta	late

**Figure 3:**
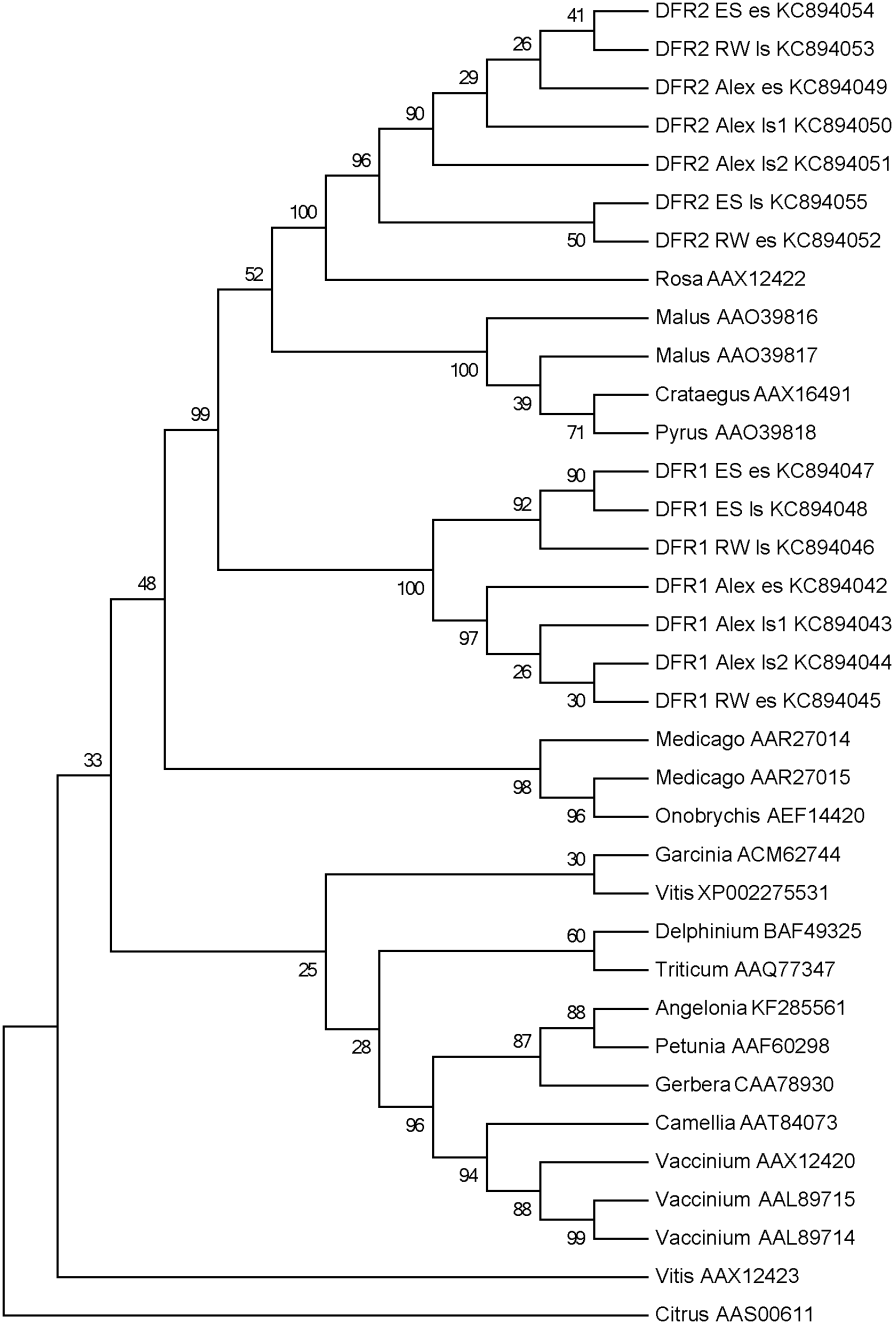
Neighbour-joining tree of *DFR* amino acid sequences of *Fragaria* and various species published in the NCBI GenBank. DFR1 *F. vesca* cv Alexandria, early stage (KC894042), late stage 1 (KC894043), late stage 2 (KC894044), cv Red Wonder early stage (KC894045), late stage (KC894046), *F*.×*ananassa* cv. Elsanta, early stage (KC894047), late stage (KC894048), DFR2 *F. vesca* cv Alexandria, early stage (KC894049), late stage 1 (KC894050), late stage 2 (KC894051), cv Red Wonder early stage (KC894052), late stage (KC894053), *F*.×*ananassa* cv. Elsanta, early stage (KC894054), late stage (KC894055), *Angelonia*×*angustifolia* (KF285561), *Camellia sinensis* (AAT84073), *Citrus sinensis* (AAS00611), *Crataegus monogynae* (AAX16491), *Delphinium belladonna* (BAF49325), *Garcinia mangostane* (ACM62744), *Gerbera hybrida* (CAA78930), *Malus*×*domestica* (AAO39816, AAO39817), *Medicago truncatula* (AAR27014, AAR27015), *Onobrychis viciifolia* (AEF14420), *Petunia*×*hybrida* (AAF60298), *Pyrus communis* (AAO39818), *Rosa hybrida* (AAX1242), *Triticum aestivum* (AAQ77347), *Vitis vinifera* (AAX12423, XP002275531), *Vaccinium macrocarpon* (AAL89715, AAL8971, AAX12420). The bootstrap values are indicated next to the relevant nodes (1000 replicates).

The phylogenetic relationship between the *DFR*s of the various *Fragaria* species was analyzed via a neighbor-joining tree that includes further amino acid sequences of *DFRs* accessible in the GenBank (Figure 3). In this tree, the *DFRs* of the Rosaceae family formed a separate cluster. Within this cluster the *DFR2s* from *Fragaria* form a group together with the *DFRs* of rose (*Rosa*×*hybrida*, AAX12422), apple (*Malus*×*domestica,* AAO39816, AAO39817), pear *(Pyrus communis* AAO39818) and hawthorn (*Crataegus monogynae,* AAX1649). *Fragaria DFRs1* and *DFRs2* are clearly revealed in different clusters with *DFR2s* more closely related to the other Rosaceous *DFR*s than to *DFR1*.

### Studies with the recombinant enzymes

The cDNA clones were transferred into a pYES expression vector and heterologously expressed in yeast (*Saccharomyces cerevisiae*). All recombinant enzymes demonstrated functional activity catalyzing the NADPH-dependent conversion of dihydroflavonols into leucoanthocyanidins. Control reactions with preparations from yeast cells harbouring the empty vector did not show DFR activity. DFR1s and DFR2s significantly differed in their acceptance of dihydroflavonols. The recombinant DFR2s converted DHQ and DHM to leucocyanidin and leucodelphinidin, but did not accept DHK as a substrate. Recombinant DFR1 s, in contrast, were selective for DHK (Figure S2 in File S1).

The two identified DFR variants were further characterized using the *DFR* pair isolated from ripe fruits of *F.*×*ananassa* cv. Elsanta. Apart from substrate acceptance, no striking differences were observed in the biochemical or kinetic characteristics of the two recombinant enzymes (Table 2). The highest reaction rates were observed in a weak acidic environment. The kinetic data, particularly the low *K*_m_ and the high *K*_cat_ */K*_m_ value, confirmed the selectivity of DFR1 for DHK. DFR2 had a higher specificity for DHQ than for DHM displaying lower *K*_m_ values and higher *K*_cat_ */K*_m_ values with DHQ as substrate (Table 2). Testing of substrates was performed under optimized conditions for the respective substrate and was within the linear range of the reaction. The selected incubation time and protein concentration ensured that the maximum conversion rate of the best substrate did not exceed 50%. As frequently observed in heterologous expression systems, the substrate selectivity was less obvious, when the protein was present in excess and incubation time was extended beyond the linear range of the reaction. The distinct substrate specificity of the DFRs, however, was confirmed in assays in which DHK and DHQ were simultaneously offered as substrates. When DHK and DHQ were present in equimolar amounts, DFR1 exclusively converted DHK to leucopelargonidin whereas in assays with the recombinant DFR2 only the formation of leucocyanidin could be observed (Figure S2 in File S1).

**Table 2:** Characterization of recombinant DFR1 and DFR2 from *Fragaria*×*ananassa* cv. Elsanta obtained from heterologous expression of *DFRs* (KC894048, KC894055) in yeast.

	*Recombinant DFR1* (KC894048)	*Recombinant DFR2* (KC894055)
pH optimum	6.00^1^	6.25^2^/5.75^3^
Temperature optimum [°C]	40	40
Temperature stability [°C]	30	30
Time linearity [min]	25	20
Protein linearity [µg]	20	25
apparent *K*_cat_ [µmol/kg*s]	11.4^1^	3.1^2^/11.2^3^
apparent *K*_m_ [µM]	0.40^1^	0.40^2^/2.3^3^
*K*_cat_ */K*_m_ [l/s*kg]	28^1^	7.3^2^/4.9^3^

using ^1^DHK, ^2^DHQ, ^3^DHM as substrates.

To date, only two variants of DFRs have been reported in the literature: non-specific DFRs, converting all types of dihydroflavonols, and specific DFRs converting only DHQ and DHM. DFR1 from *Fragaria* species represents a third variant of DFR, which prefers DHK. The simultaneous presence of several *DFR* genes has been demonstrated in several plant species [15], [16], [17], [18], [19]. It is important to note that gene copies may encode enzymes with different substrate specificities. Drawing conclusions on the substrate specificity of a DFR just from the observed flavonoid hydroxylation pattern should therefore be avoided.

### Sequence analysis

DFR1 and DFR2 sequences were analyzed for systematic differences that might be deciding factors for the differing substrate acceptance. The translated amino acid sequences demonstrated the highest divergence at both the N- and C-terminus (Figure 2). DFR2 of both *Fragaria* species had 349 aa and was generally longer than DFR1, which had 341 aa in *F*.×*ananassa* and only 333 aa in *F. vesca*. Apart from the variable termini, the alignment of the sequences from aa 6–330 (numbering according to DFR1) revealed 36 points of distinct differences. 12 of the differences are located in a region, which was identified by Johnson et al. [10] as being relevant for determining substrate specificity (aa 126–170 in the gerbera DFR corresponding to 128–172 in DFR1). Position 134 in the gerbera DFR is of particular interest in this regard. The presence of an aspartic acid in the petunia DFR sequence, which contrasts with the more frequently occurring asparagines, was suggested to determine the inability of converting DHK [10]. In addition, the exchange of the asparagine in the gerbera DFR into a non-polar leucine converts the non-specific DFR into a ‘DHK specialist’ [10]. The crystal structure obtained from a recombinant *Vitis vinifera* DFR confirmed the importance of this position (in this case aa 133). Asparagine 133 coordinates the dihydroflavonol substrate via interaction with the hydroxyl groups in position 3′ and 4′ [20]. In the DFRs from strawberry, amino acid 133 is an asparagine in DFR2 but an alanine in DFR1. The presence of a non-polar amino acid in the DHK-specialist is in line with [10]. The observed substrate specificity of DFR2, however, cannot be explained by the presence of the asparagine, because this is also found in the non-specific DFR from gerbera.

### Relevance of DFR for the anthocyanin hydroxylation pattern in *Fragaria* species

**Figure 4:**
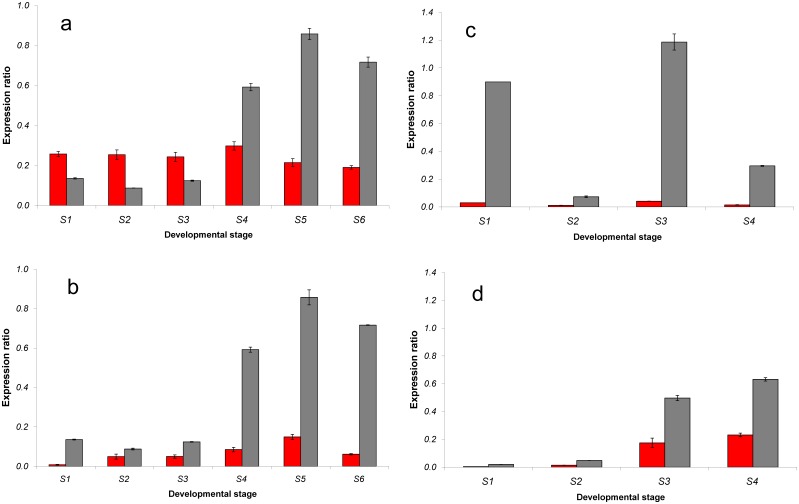
Quantitative expression of *DFR1* and *DFR2* normalized to *glyceraldehyde 3-phosphate dehydrogenase* in receptacle and achenes of *Fragaria* fruits during the different stages of fruit development. a: *F.*×*ananassa* receptacle, b: *F.*×*ananassa* achenes, c: *F. vesca* receptacle, d: *F. vesca* achenes. red: *DFR1*, grey: *DFR2.* Data were calculated from three biological replicates with at least two technical replicates for each and with error bars representing the standard deviation.

**Figure 5:**
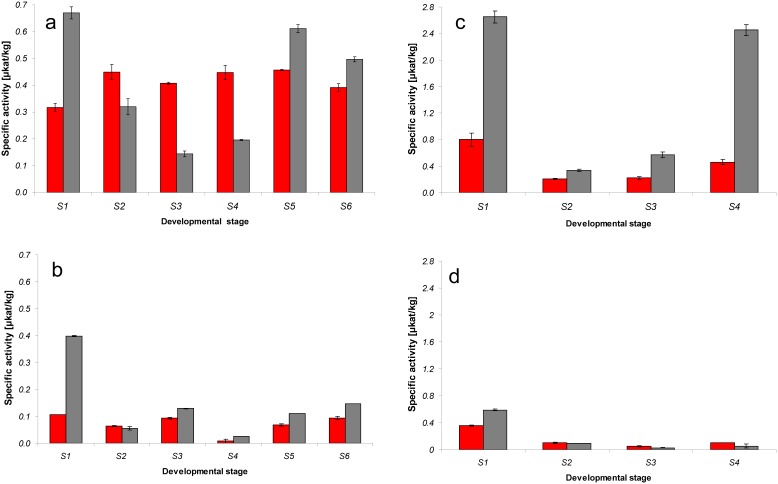
Specific DFR activity [nkat/kg protein] with DHQ and DHK as substrates in receptacle and achenes throughout the different stages of fruit development and ripening in *F.*×*ananassa* and *F. vesca*. a: *F.*×*ananassa* receptacle, b: *F.*×*ananassa* achenes, c: *F. vesca* receptacle, d: *F. vesca* achenes. red: *DFR1*, grey: *DFR2.* Data were calculated from three biological replicates and with error bars representing the standard deviation.

The expression profiles of *DFR1* and *DFR2* were studied in the strawberry fruits. Botanically they are not berries but aggregate fruits, where the so-called ‘seeds’ are the real *Fragaria* fruits (achenes). The edible part which is commonly referred to as the fruit stems from the receptacle. The polyphenol profile and proteome varies between the tissues [21], [22], [23]. For this reason, receptacles and achenes were studied separately. The quantitative Real-time PCR data for the *DFRs* were normalized against *actin* (Figure S3 in File S1) and *glyceraldehyde 3-phosphate dehydrogenase* (*GAPDH*) (Figure 4). With both housekeeping genes comparable results were obtained with only slight differences for the early stages of the *F*.×*ananassa* receptacle (Figure 4). In the receptacle of both species, the profile of *DFR2* expression fluctuated during fruit development while for *DFR1* expression this was less pronounced. In the achenes, the *DFR* expression increased during fruit ripening in both species. In *F. vesca, DFR2* expression was drastically higher than the *DFR1* expression in both tissues during all developmental stages and showed two maxima for the receptacle during stage 1 and 3 with a decline in stage 2 (Figure 4). The highest *DFR1* expression in the *F. vesca* fruits was observed in S3 and S4 of the achenes. In *F*.×*ananassa, DFR2* expression was higher in late developmental stages of the fruits, whereas *DFR1* expression remained near stable along the different stages of fruit development and ripening. In the first three stages, *DFR1* expression was higher than *DFR2* expression, in the later stages the ratio reversed, but *DFR1* expression was still significantly higher than in the *F. vesca* receptacle (Figure 4). This was reflected by a differing substrate acceptance of enzyme preparations obtained from strawberry fruits (Figure 5). Preparations from *F. vesca* receptacles demonstrated higher specific activity with DHQ other than with DHK in all the developmental stages of the fruit. Preparations from *F*.×*ananassa* displayed variable dihydroflavonol preference during fruit development with higher specific activity with DHK as a substrate compared with DHQ in S1-S3, and still lower ones in S4-S6. DHK acceptance of preparations from *F*.×*ananassa,* however, was always higher than with preparations from *F. vesca*, even in the late developmental stages (Figure 5). Profiles of gene expression and enzyme activities were not always consistent. This was particularly the case in the achenes obtained from S3 and S4 fruits of *F. vesca* with drastically lower enzyme activity observed than what could have been expected from the high *DFR* expression levels. We assume, however, that this could be a problem related to increased levels of polyphenols as well as proteins and lipids in the achenes which might hamper the enzyme activity measurements. In *F. ananassa*, this discrepancy was observed to a much lower extent, which is in line with the fact that wild species frequently show increased levels of polyphenols in comparison to their domesticated counterparts [1]. To contrast with S1 and S2 receptacles of *F*.×*ananassa DFR* expression was relatively low compared to enzyme activity. Harmonized profiles, however, can not necessarily be expected. The life span of the DFR enzymes in the cell is completely unknown, and observed activity could be a cumulative result of DFRs produced during different stages, possibly resulting in a shift of maxima between stages. It cannot even be excluded that DFR activities in very early stages result partially from *DFR* expression in the flowering period. In addition to this, post-transcriptional regulation may play a role.

It would appear that DFR does help to determine the pattern of flavonoid hydroxylation in strawberry fruits. In *F. vesca*, a high *F3'H* expression during all developmental stages continuously promotes the availability of flavonoids with a 3′,4′-dihydroxylation pattern including DHQ which is converted into leucocyanidin by the enzyme encoded by the highly expressed *DFR2*. As leucocyanidin is the immediate precursor to the formation of dark-red cyanidin based pigments, this results in a drastically higher cyanidin:pelargonidin ratio in the *F. vesca* fruits (0.51) compared to *F.*×*ananassa* (0.05). Total anthocyanin concentrations were, however, with 125 mg/kg lower than in *F.*×*ananassa* (350 mg/kg). We assume, however, that in *F. vesca* an additional factor could be relevant as the observed concentrations of pelargonidin based pigments are higher than would have been expected from the high levels of *DFR2* and *F3'H* transcripts. Measurements of the DFR activity during the four developmental stages of the *F. vesca* receptacle with DHQ and DHK as substrates confirmed a high DFR activity with a persistent DHQ acceptance in all stages (Figure 5). The relatively high DHK acceptance compared to the low *DFR1* expression level is in line with the higher enzyme efficiency compared to DFR2 (Table 2). In addition, it is possible that F3*'*H activity in the *F. vesca* fruits is lower than expected from the high transcript levels. However, this could not be verified by enzyme assays as the F3*'*H is a membrane associated enzyme and is difficult to measure in tissues which are rich in disturbing compounds such as polyphenols, sugars and glucanes [24]. In *F.*×*ananassa,* the observed transcript levels are in line with the anthocyanin composition of the fruits. Due to the decrease of *F3'H* transcript levels during fruit ripening, a depletion of 3′,4′-hydroxylated dihydroflavonols is expected which prevents the formation of large amounts of cyanidin, despite the high transcript levels of *DFR2*. The relatively high DFR1 expression levels compared to *F. vesca* and the high enzyme efficiency of DFR1 demosntrated by the high *K*_cat_ */K*_m_ values, however, provides sufficient amounts of precursors to allow the formation of leucopelargonidin as the immediate precursors for pelargonidin formation.

### Conclusions

Our studies identified a novel DFR variant in *Fragaria* sp., which demonstrates an unusual DHK preference. The two DFR variants show divergent expression profiles in both of the two species but also with regards to the fruit development. It is likely that these expression profiles are due to differences in the transcript regulation of the two genes. The higher expression rate of DFR1 and the higher enzyme efficiency of DFR1 is an important precondition for the accumulation of pelargonidin based pigments in *F.*×*ananassa* together with the absence of F3*'*H expression in the late stages. In *F. vesca*, a high *DFR2* and *F3'H* expression account for the increased levels of cyanidin based pigments. The novel DFR pair derived from a single species will be a perfect model for future studies into the molecular background behind DFR substrate specificity.

## Materials and Methods

### Plant material

The *s*tudies were performed on fruits of *Fragaria*×*ananassa* cv. Elsanta harvested at the experimental orchard of the Institute of Horticulture and Viticulture (University of Natural Resources and Applied Life Sciences, Vienna, Austria), and *F. vesca* cv. Red Wonder and cv. Alexandria grown at JKI Dresden-Pillnitz, Germany. The fruits were shock-frozen in liquid nitrogen and stored at −80°C. The samples were identical to those recently used for the studies on F3*'*H in strawberry [25]. Briefly, the six developmental stages of *F.*×*ananassa* were small-sized (0.7 cm) green fruits (S1), middle-sized (1.5 cm) green fruits (S2), middle-sized (2–2.5 cm) white fruits (S3), middle-sized (2–2.5 cm) turning-stage fruits (S4), middle-sized (2–2.5 cm) late turning-stage fruits (S5), and full-ripe red fruits, 4 cm fruit size (S6). The four developmental stages of *F. vesca* comprised small-sized (0.3 cm) green fruits (S1), middle-sized (0.5 cm) turning-stage fruits (S2), late turning-stage fruits with 0.6 cm fruit size (S3) and full-ripe red fruits of 0.8 cm fruit size (S4) [25]. Due to differences in morphology and fruit development, stages of fruit development between the two species were not defined in a similar way. It is possible, however, to distinguish between unripe fruits (*F. vesca* S1 and *F*.×*ananassa* S1+2), turning stage fruits (*F. vesca* S2+3 and *F*.×*ananassa* S4+5) and ripe fruits (*F. vesca* S4 and *F*.×*ananassa* S6). Receptacle and achenes were separated manually from frozen fruits without defrosting the material. The tissues were analyzed separately for enzyme activity and gene expression.

### Chemicals

(2-^14^C)-Malonyl-coenzyme A (55 mCi/mmol) was purchased from New England Nuclear Corp. GmbH (Vienna, Austria). (^14^C)-Labeled flavonoids DHK, DHQ, and DHM were synthesized as previously described [26], [27] using recombinant F3*'*5*'*H from *Sollya heterophylla* and F3*'*H from *Tagetes erecta*.

### Cloning of DFR cDNAs from Fragaria species

mRNA was isolated from strawberry fruits with µMACS mRNA Isolation Kit (Miltenyi Biotec, Germany). cDNA was prepared using the SuperScript II Reverse Transcriptase (Invitrogen, Carlsbad, CA) and the oligo(-dT) anchor primer GACCACGCGTATCGATGTCGAC(T)_16 _V. The genome of *F. vesca* was screened for sequences with high similarity to a DFR from *F*.x *ananassa* (AY695812) using the tools available at www.rosaceae.org.

Specific primers (DFR1_f: ATGGGGTTGGGAGCTGAATC, DFR1_r: TCAACCAGCCCTGCGCTTT, DFR2_f: TGTCAAGAAACATGGGATCGGAG, DFR2_r: GAAGCTCTCAACATACAGAAGATAGA) were designed on the basis of the detected sequences. Proof reading PCR was carried out using the Expand High Fidelity Plus PCR System (Roche, Austria) according to the manufacturer’s instructions. PCR products were ligated into the vector pYES2.1/V5-His-TOPO and transformed into *E. coli* strain Top 10 (Invitrogen, Carlsbad, CA) according to the manufactureŕs instructions. Plasmids were isolated with Wizard Plus SV Minipreps DNA Purification System (Promega, Vienna, Austria) and sequenced by a commercial supplier (Microsynth Austria AG, Vienna, Austria).

### Gene expression analysis

The expression of the *DFRs* was quantified by qPCR using a StepOnePlus system (Applied Biosystems, Darmstadt, Germany) and the SybrGreenPCR Master Mix (Applied Biosystems, Vienna, Austria) according to the supplier*’*s instruction. The analysis was carried out in three independent triplicates, and the data was normalized against two control genes, actin and glyceraldehyde 3-phosphate dehydrogenase (*GAPDH*). The relative expression ratio of a target gene was computed applying the equation according to [28]. The efficiency of the PCR-reaction was determined on the basis of standard curves which were obtained by applying different DNA concentrations. The product specificity was confirmed via melt curve analysis and gel electrophoresis. Primers for *DFRs* were designed for the isolated *DFR* sequences (Table S1 in File S1).

### Sequence and Phyologenetic Analysis

Multiple alignments were undertaken with Clustal Omega [29], [30]. The phylogenetic tree was conducted and bootstrapped with MEGA version 5 [31] using the neighbor-joining method and 1000 replicates.

### Anthocyanin determination

For the determination of the anthocyanin content, 10 g of shock-frozen petals were pulverized and mixed with 35 ml 2 M methanolic hydrochloric acid. After shaking for 12 hours at 4°C in an overhead rotator, the suspension was centrifuged for 10 minutes at 19200×g. 10–140 µl of the supernatant was adjusted with 2 M methanolic hydrochloric acid to a final volume of 1000 µl. The absorption at 520 nm was determined on a DU-65 spectrophotometer (Beckman Instruments). The anthocyanin content was calculated as pelargonidin equivalent using a calibration curve obtained with commercially available pelargonidin chloride (Roth, Germany). For acidic hydrolysis of anthocyanins, 20 µl methanolic hydrochloric acid extract were mixed with 180 µl of 4 N HCL and incubated for 60 minutes at 90°C. After cooling for 10 minutes the mixture was centrifuged for 10 minutes at 10000×g. The supernatant was adjusted to 200 µl with 4 N HCL and aliquots were used for HPLC analysis [32] using a Perkin Elmer Series 200 HPLC system equipped with a Perkin Elmer Series 200 diode array detector and Total Chrom Navigator, version 6.3.1 (Perkin Elmer Inc). The column was a BDS Hypersil C18 HPLC column, 5 µm, 250×4.6 mm (Thermo Scientific).

### Heterologous expression and protein preparation

The vectors harbouring the *DFR* cDNAs were transformed into yeast strain INVSc1 using the Sc. EasyComp Transformation Kit (Invitrogen, Carlsbad, CA). Preparation of the protein fractions was performed using a modified protocol according to Pompon et al. [33]. Briefly, 250 ml of expression culture was grown in YPGE medium (5 g/l glucose, 10 g/l peptone, 10 g/l yeast extract, 30 ml 100% ethanol) at 28°C and 180 rpm for approximately 6 h, until an OD_600_ of 0.8–1.2 was reached. After addition of 27 ml 20% (w/v) sterile filtered galactose, the culture was incubated at 28°C and 180 rpm for 15 h. Cells were harvested by centrifugation at 4000× g at 4°C for 10 min, with TEK (50 mM Tris/HCl, 1 mM EDTA, 100 mM potassium chloride, pH 7.4), and redissolved in 2.5 ml icecold buffer TES-B* (50 mM Tris/HCl, 1 mM EDTA, 0.6 M sorbitol, 2 mM dithiothreitol, pH 7.4). Disruption of cell walls was achieved via vigourous shaking with glass beads for 30 s every minute during a period of 20 min. Glass beads were removed by centrifugation at 4000×g and 4°C for 10 min. The protein preparation was diluted with 2.5 ml icecold buffer TES-B*, shock frozen in liquid nitrogen and stored at −80°C.

### Enzyme preparation

Enzyme preparations from strawberry fruits were obtained by using the protocol of Claudot and Drouet [34] with slight modifications as previously described [24]. To remove low molecular weight compounds, crude enzyme preparations were passed through a gel chromatography column (Sephadex G25, GE Healthcare, Freiburg, Germany). Protein content was determined by a modified Lowry procedure [35] using crystalline bovine serum albumin as a standard.

### Enzyme assays

Assays for DFR were performed as described earlier [27], [36]. Briefly, the reaction contained in a final volume of 50 µl: 1–10 µl enzyme preparation (2.4–24 µg), 0.048 nmol (^14^C)-dihydroflavonol, 0.25 nmol NADPH, 44–35 µl 0.1 M KH_2_PO_4_/K_2_HPO_4_ buffer pH 6.3 containing 0.4% Na ascorbate. The amount of enzyme preparation depended on the recombinant enzyme used and was chosen to ensure a maximum conversion rate with the best substrate at 50%.

### Enzyme characterization

All data represents an average of at least three independent experiments. Determination of the pH optimum was carried out as described for the standard DFR assay, but using 0.2 M McIlvaine buffers with pH values between 4.5 and 9.0. Optimal temperature was determined by measuring activities at varying temperatures within 0°C and 60°C. Temperature stability was determined by measuring enzyme activities at 25°C after incubation of the reaction mixture without NADPH at varying temperatures. Kinetic data were calculated from Lineweaver-Burk plots using radiolabeled substrates at varying concentrations.

## Supporting Information

File S1 **File includes Figures S1–S3 and Table S1.** Figure S1: Left: Ripe strawberry fruits of *F. vesca* cv. Red Wonder. Right: *F.*×*ananassa* cv. Elsanta. Due to differing magnification factors used, fruit size does not appear at a comparable scale. Figure S2: Radioscan of TLC on cellulose from incubation of recombinant DFR2 (left) and DFR1 (right) in the presence of NADPH offering A: (^14^C)dihydroquercetin, B: (^14^C)dihydrokaempferol, C and D: (^14^C)dihydroquercetin and (^14^C)dihydrokaempferol in equimolar amounts as substrates. Figure S3: Quantitative expression of *DFR1* and *DFR2* normalized to *actin* in receptacle and achenes of *Fragaria* fruits along the different stages of the fruit development. a: *F.*×*ananassa* receptacle, b: *F.*×*ananassa* achenes, c: *F. vesca* receptacle, d: *F. vesca* achenes. red: *DFR1*, grey: *DFR2.* Data were calculated from three biological replicates with at least two technical replicates for each and error bars representing the standard deviation. Table S1: List of primers used for quantitative Real-time PCR.
(DOC)
