# Supplementary material for: Dihydroflavonol 4-Reductase Genes Encode Enzymes with Contrasting Substrate Specificity and Show Divergent Gene Expression Profiles in Fragaria Species
Source: PLoS One. 2014 Nov 13;9(11):e112707. doi: 10.1371/journal.pone.0112707 (PMC4231056; doi:10.1371/journal.pone.0112707)
Supplement: File S1 — File includes Figures S1–S3 and Table S1. Figure S1: Left: Ripe strawberry fruits of F. vesca cv. Red Wonder. Right: F.×ananassa cv. Elsanta. Due to differing magnification factors used, fruit size does not appear at a comparable scale. Figure S2: Radioscan of TLC on cellulose from incubation of recombinant DFR2 (left) and DFR1 (right) in the presence of NADPH offering A: (14C)dihydroquercetin, B: (14C)dihydrokaempferol, C and D: (14C)dihydroquercetin and (14C)dihydrokaempferol in equimolar amounts as substrates. Figure S3: Quantitative expression of DFR1 and DFR2 normalized to actin in receptacle and achenes of Fragaria fruits along the different stages of the fruit development. a: F.×ananassa receptacle, b: F.×ananassa achenes, c: F. vesca receptacle, d: F. vesca achenes. red: DFR1, grey: DFR2. Data were calculated from three biological replicates with at least two technical replicates for each and error bars representing the standard deviation. Table S1: List of primers used for quantitative Real-time PCR. (DOC) [file pone.0112707.s001.doc]

**Table S1: List of primers used for quantitative Real-time PCR.**

| **Primer Name** | **Sequences (5´>3´- direction)** | **T_m_ (°C)** |
| --- | --- | --- |
| Fra.Actin.q.for | TCGTGTTGCCCCAGAAGAGC | 61.4 |
| Fra.Actin.q.rev | CACGATTAGCCTTGGGATTCAG | 60.3 |
| Fra.GAPDH.q.F1 | AGACATCTGCTGGAGTTACCAC | 61.3 |
| Fra.GAPDH.q.R1 | CATTGAGAGCAGGCAGAACCTTT | 60.6 |
| Fra.DFR1.q F1 | agacatgctttcatgatgtctaaca | 60.3 |
| Fra.DFR1.q.R1 | CTTCGCTGTGGAAGGCGGACCTGGACGT | 60.3 |
| Fra.DFR2.q F1 | AGCATCTGCTAGACTTGCCGA | 59.8 |
| Fra.DFR2.q.R1 | GGCTTTTGGGTCTCTTCAACATTT | 59.3 |

Figure S1: Left: Ripe strawberry fruits of *F. vesca* cv. Red Wonder. Right: *F.* x *ananassa* cv. Elsanta. Due to differing magnification factors used, fruit size does not appear at a comparable scale.

Figure S2: Radioscan of TLC on cellulose from incubation of recombinant DFR 2 (left) and DFR1 (right) in the presence of NADPH offering a: (^14^C)dihydroquercetin, b: (^14^C)dihydrokaempferol, c and d: (^14^C)dihydroquercetin and (^14^C)dihydrokaempferol in equimolar amounts as substrates


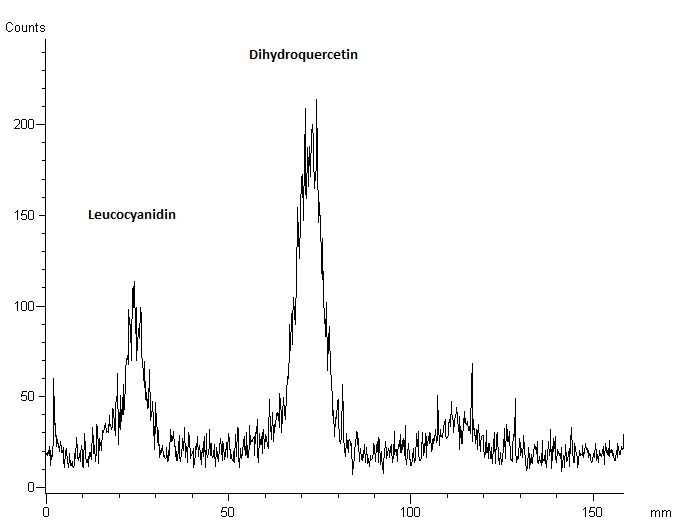

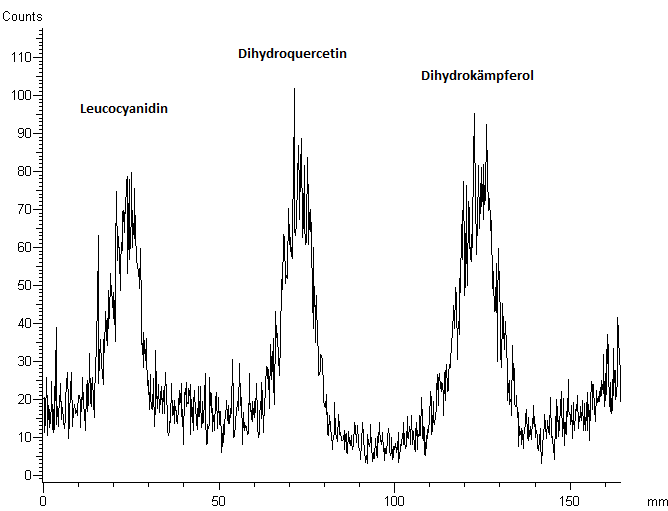

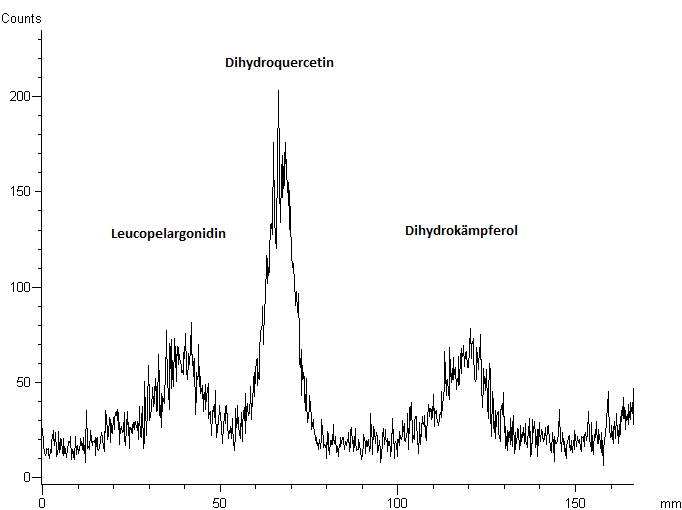

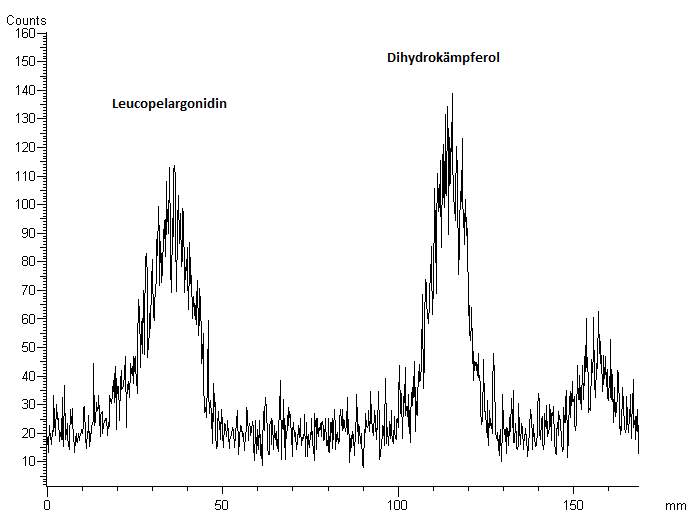


**Dihydrokaempferol**

**Dihydrokaempferol**

**Dihydrokaempferol**

a

b

c

d

Figure S3: Quantitative expression of *DFR1* and *DFR2* normalized to *actin* in receptacle and achenes of *Fragaria* fruits along the different stages of fruit development. a: *F.* x *ananassa* receptacle, b: *F.* x *ananassa* achenes, c: *F. vesca* receptacle, d: *F. vesca* achenes.

red: *DFR1*, grey: *DFR2.* Data were calculated from three biological replicates with at least two technical replicates for each and error bars represent the standard deviation.
